# Novel Markers in Diabetic Kidney Disease—Current State and Perspectives

**DOI:** 10.3390/diagnostics12051205

**Published:** 2022-05-11

**Authors:** Agnieszka Piwkowska, Łukasz Zdrojewski, Zbigniew Heleniak, Alicja Dębska-Ślizień

**Affiliations:** 1 Laboratory of Molecular and Cellular Nephrology, Department of Molecular Biotechnology, Faculty of Chemistry, Mossakowski Medical Research Institute Polish Academy of Sciences, University of Gdansk, 80-308 Gdansk, Poland; apiwkowska@imdik.pan.pl; 2 Department of Nephrology, Transplantology, and Internal Medicine, Faculty of Medicine, Medical University of Gdansk, 80-308 Gdansk, Poland; zbigniew.heleniak@gumed.edu.pl (Z.H.); adeb@gumed.edu.pl (A.D.-Ś.)

**Keywords:** diabetic kidney disease, diabetes, novel markers

## Abstract

Diabetic kidney disease (DKD) is a leading cause of end-stage renal disease. Along with the increasing prevalence of diabetes, DKD is expected to affect a higher number of patients. Despite the major progress in the therapy of DKD and diabetes mellitus (DM), the classic clinical diagnostic tools in DKD remain insufficient, delaying proper diagnosis and therapeutic interventions. We put forward a thesis that there is a need for novel markers that will be early, specific, and non-invasively obtained. The ongoing investigations uncover new molecules that may potentially become new markers of DKD—among those are: soluble α-Klotho and proteases (ADAM10, ADAM17, cathepsin, dipeptidyl peptidase 4, caspase, thrombin, and circulating microRNAs). This review summarizes the current clinical state-of-the-art in the diagnosis of DKD and a selection of potential novel markers, based on up-to-date literature.

## 1. Introduction and Epidemiology

DM is a metabolic disease characterized by hyperglycemia that results from improper insulin secretion and/or target tissues resistance to insulin. Long-lasting and recurrent hyperglycemia leads to the injury, dysfunction, and failure of internal organs, including the kidneys, thus causing DKD. 

A developing epidemic of this metabolic disease is expected to four-fold increase the number of patients with DM, comparing the 1980s to the 2030s. The number of patients, estimated at 422 million in 2014, is expected to rise to 590 million by 2035 (according to the World Health Organization Report) [1,2]. Up to 40% of all individuals with DM will develop DKD, which will direct them to a path of progressive chronic kidney disease (CKD) and further increase their risk of end-stage renal disease (ESRD), cardiovascular diseases (CVD), and CVD-related death [3]. Progressive DKD is a leading reason for ESRD and the need for cost-consuming renal replacement therapies, both in Europe and the United States [4,5]. 

## 2. Diagnosis

The straightforward definition of DKD is a chronic kidney disease with DM, resulting in hyperglycemia as the primary reason for kidney injury. Therefore, the classical clinical diagnosis is made bidirectionally. First, patients are screened for the presence of albuminuria, which is the lowest range proteinuria that can be found in the course of DKD. It is usually expressed as a fraction of the spot urine albumin-to-creatinine ratio. Values of ≥30 mg/g are pathologic, and this value needs to be confirmed in at least two out of three trials, made over 3 to 6 months.

Secondly, a regular assessment of serum creatinine concentration and the resulting estimation of the glomerular filtration rate (eGFR) value is made, with its pathologic values of <60 mL/min/1.73 m^2^ or higher, when accompanied with albuminuria.

The above-mentioned allow for assigning an individual patient to a specific stage of CKD, according to given definitions, e.g., KDIGO (kidney disease improving global outcome) guidelines [6]. To increase the specificity of these criteria, clinicians take into account the presence of other diabetes-derived microangiopathies (e.g., diabetic retinopathy) and time that elapsed since the diagnosis of DM.

As can be clearly seen, DKD, when defined this way, is an indirect diagnosis, or a diagnosis of exclusion of other CKD etiologies. Firstly, there is always potentially a reason other than DKD for progressing renal failure with accompanying proteinuria (e.g., primary glomerulonephritis, atherosclerotic nephropathy, and paraproteinemia). Secondly, even though there is a well-known correlation between the length of DM and prevalence of proteinuria on the one hand, and the resulting correlation between proteinuria and the prevalence of renal failure on the other, we still miss a specific cut-off value for an individual patient [7]. There is neither a consensus on how long diabetes should be present in an individual to cause DKD, nor what the cut-off lines of proper glycemic control are, behind which the risk of DKD development rises. Thirdly, the clinical trajectories of DKD, that differ from classical one, will most probably avoid detection, especially at its early stages. To conclude, there are currently no reliable biomarkers for the early detection of impaired kidney function, which can enable therapeutic interventions to prevent or slow disease progression. Therefore, the theoretical golden standard in DKD should be the core needle biopsy, with its potential risks as an invasive procedure.

## 3. Biopsy and Histopathology of DKD

Kidney biopsy remains a basic diagnostic tool in the hands of clinical nephrologists. Still remaining a golden standard in DKD diagnosis, it is relatively rarely performed. Its main drawbacks are the relatively frequent complications (e.g., hematomas 11%, pain at the site 4.3%, and hematuria 3.5%) and possibility of more serious undesirable outcomes, such as bleeding requiring blood transfusions (1.6%), interventions to stop bleeding (0.3%), or death (0.06%) [8]. Therefore, being capable of providing clinicians with early and certain diagnosis, kidney biopsy in the course of DM is reserved only to clinically controversial cases (in the absence of retinopathy, sudden onset of proteinuria or nephrotic syndrome, presence of hematuria, and rapid decline of renal function).

Following the biopsy, light microscopy reveals characteristic glomerular changes in the course of diabetic nephropathy. They mostly consist of mesangial expansion that, in its most pronounced form, creates nodular lesions, such as the typical Kimmelstiel–Wilson nodule thickening of the glomerular basement membrane. Pathologic classifications of diabetic nephropathy concentrate on the stage of morphologic lesions, assessing the degree of mesangial expansion, in relation to capillary lesions (stages I—III), and presence of Kimmelstiel–Wilson nodules (stage III), ending at most advanced sclerosis of the glomerular vascular pole (stage IV by Tervaert et al.) [9,10]. The glomerulus is the primary site of diabetic injury in the kidney. Glomerular hypertrophy and podocyte depletion are glomerular hallmarks of progressive DKD, and the degree of podocyte loss correlates with the severity of the disease. Podocytes are highly specialized cells that wrap around glomerular capillaries. They comprise of a key component of the glomerular filtration barrier. Podocytes consist of three morphologically and functionally different segments: a cell body, major processes, and foot processes. The podocyte cell body gives rise to primary processes that branch into foot processes; in turn, the foot processes (FPs) of neighboring podocytes establish a highly branched, interdigitating pattern, known as a slit diaphragm. The slit diaphragm represents a signaling platform that regulates podocyte function and consists of many proteins, such as nephrin, podocin, Neph1, insulin receptor, and actin [11].

The podocyte slit diaphragms are the target of injury in many glomerular diseases, including arterial hypertension, inflammation, and DM.

Studies in patients with microalbuminuric type 1 diabetes demonstrated an increase in the width of podocyte foot processes, compared with that in the podocytes of healthy individuals. The width of foot processes was shown to directly correlate with the urinary albumin excretion rate. In addition, the number and density of podocytes have been reported to be markedly reduced in patients with either type 1 or 2 diabetes [12,13]. Therefore, exploring the mechanism of cell injury in DKD and finding new therapeutic targets and biomarkers may be helpful in distinguishing the patients at risk of kidney failure from those who are likely to recover function.

## 4. Classic and Alternative Clinical Patterns of DKD

In recent years, it has been argued that the actual clinical course of DKD is not unidirectional and differs, in terms of albuminuria and proteinuria presence or dynamics of GFR loss, thus impeding the differential diagnosis of nephropathy [3,14].

### 4.1. Classical (Albuminuria-Based) DKD Pattern

As is well-described above, with a natural course starting with hyperfiltration and renal hypertrophy (stages 1–2 by Mogensen), turning into incipient nephropathy with albuminuria (stage 3), after approximately 15 years, progressing to overt nephropathy with proteinuria, nephrotic syndrome, and accelerated renal failure progression (stage 4), finally reaching end-stage kidney disease after approx. 25 years [15].

### 4.2. Non-Albuminuric DKD Pattern

The non- albuminuric DKD pattern is characterized by the decreased renal function, with eGFR < 60 mL/min/1.73 m^2^ in diabetic patients with normoalbuminemia (uACR < 30 mg/g). The prevalence of this condition among DM type 2 patients is assessed at 14–28% [16,17]. This condition carries a lower risk for ESKD, CKD progression, or a rapid decline in eGFR, when compared with the classical pathway. Despite the lower risk, in general, reports show that a number of individuals with a non-albuminuric pattern show faster progression to advanced kidney failure, in comparison to the classic pathway. However, we still miss a diagnostic tool to identify this at an early stage.

### 4.3. “Regression of Albuminuria” Pattern

As proved in Japanese and Danish populations, the appearance of albuminuria is not unidirectional, and a regression of albuminuria is present in 30–51% of patients [18,19]. Apart from natural factors, such as glycemic control improvement, renin–angiotensin–aldosterone blockage therapies and (probably) SGLT-2 inhibition will add to the regression of albuminuria, thus reducing renal function loss [20].

### 4.4. “Early Rapid Decliner” Pattern

An annual decline in eGFR of ≥5 mL/min/1.73 m^2^ is claimed as “rapid”, according to KDIGO guidelines nomenclature, and clearly higher than the average annual eGFR fall in type 2 diabetics [21]. This condition appears as independent from the albuminuria/proteinuria pattern. In a report presented by Yoshida et al., in a specific hospitalized cohort, the group of early rapid decliners reached 14% [22].

Considering the aforementioned, it is clearly seen that the widely used clinical criteria for DKD diagnosis fails to recognize anything other than the typical patterns of the disease. This results in delayed diagnosis, delayed introduction of nephroprotection, and intensification of hypoglycemic treatment. Another challenge is the differential diagnosis of DKD versus other nephropathies. The mentioned clinical patterns can disguise themselves as glomerulonephritis, tubulointerstitial diseases, or atherothrombotic nephropathies. Invasive kidney biopsy has its limited application possibilities. Therefore, there is a practical clinical need for novel DKD markers to bring early and reliable diagnosis to both clinicians and diabetic patients.

Multidirectional, physiopathological mechanisms of DKD gave birth to a wide list of potential protein markers, which are currently under investigation. While this search continues, we would like to present potential candidates that are of our special interest.

## 5. Novel Biomarkers: A Selection and Future Perspectives

### 5.1. Soluble α-Klotho

α-Klotho (αKL) was named after the Greek goddess spinning the thread of life and discovered in 1997 as a gene regulating aging [23].

α-Klotho is a transmembrane protein with a large extracellular domain consisting of the two domains, K1 and K2, which share sequence homology with β-glycosidases. This protein exists in three isoforms, a transmembrane form, shed soluble form containing K1 and K2, and truncated soluble form consisting of the K1 domain. Soluble Klotho is present in blood, urine, and cerebrospinal fluid. αKL has been proposed to function as a hormone in its soluble form, glycosidase, and coreceptor for FGF23 and FGF receptor (FGFR) in its transmembrane form [24].

The kidney is the principal contributor to circulating Klotho and, simultaneously, the major organ where circulating Klotho is taken up from the circulation [25]. The soluble α-Klotho acts, from the urinary luminal side of nephron, as an autocrine or paracrine enzyme to regulate transporters and ion channels. Klotho is mainly expressed in proximal renal tubule and the distal tubular epithelium [26]. However, it was recently demonstrated that the podocytes are another source of Klotho expression [27]. What is more, the administration of recombinant Klotho protected against the slit diaphragm disruption induced by the overexpression of TRPC6 in mice [28]. It has also been proven that soluble Klotho traffics across renal tubules, from basolateral to intracellular space, and is then secreted across the apical membrane into the urinary lumen [25].

Thus, kidney disease is expected to affect both the production and clearance of α-Klotho in a complex manner.

Reduced serum and urinary α-Klotho levels are observed in the early stages of chronic kidney disease, with a progressive decline in the more advanced stages [29,30]. Reduced blood Klotho concentration is also associated with increased albuminuria, especially in patients with diabetes [31]. The same studies argued that αKL levels may be able to mediate insulin metabolism through the inhibition of intracellular insulin signaling [32]. This indicated that a disturbance in mineral and bone metabolism seems to play a role in the pathogenesis of insulin resistance. Moreover, it appears reasonable to consider αKL as a potential biomarker and target for the early intervention and treatment of CKD and DKD (Table 1).

### 5.2. Proteases as Novel Circulating Biomarkers

It has recently become evident that podocytes are highly dynamic cells. The development of foot processes effacement, as well as the recovery from this condition, requires a high degree of podocyte motility, which involves changes in slit diaphragm structure and function, reorganization of the podocyte actin cytoskeleton, and alterations in the adhesion of podocytes to the glomerular basement membrane (GBM). All of these three aspects of podocyte dynamics are regulated through several enzymatic pathways, including signaling by phosphorylation, dephosphorylation, and proteolytic regulatory cascade [33,34,35,36,37].

Podocyte proteases regulate the glomerular response to numerous chemical, mechanical, and metabolic cues. These proteases form a protein signaling network that integrates stress stimuli and serves as a key controller of the glomerular microenvironment. Both extracellular and intracellular proteolytic networks are perturbed in focal segmental glomerulosclerosis, as well as in hypertension and DKD [33,38] (Table 1 and Table 2).

#### 5.2.1. ADAM10 and ADAM17

The cleavage and shedding of membrane proteins is an important regulatory pathway in many normal and pathological processes. It was demonstrated on cultured cells and kidney tissue slides that α- (ADAM10 and ADAM17) and β-secretase modulate αKL shedding and production of soluble αKL protein by acting on two cleavage sites: first, close to the transmembrane domain; second, between the KL1 and KL2 domains [25]. There is evidence that ADAM10 and ADAM17 play important roles in the pathogenesis of DKD [39,40,41,42,43,44]. It has been shown that glucose induces the activation of ADAM17, regulates profibrotic TGFβ, and causes the accumulation of matrix proteins in mesangial cells. Moreover, it was demonstrated that ADAM17 expression and activity are increased in the kidney cortex of OVE26 mice with type 1 diabetes. In that model, ADAM17 contributes to matrix protein accumulation, through the activation of NOX4 subunits of NADPH oxidase [39]. It was also demonstrated that ADAM10 is expressed in podocytes and displays its increased levels in the urine of patients with various glomerular diseases [43]. The activity of ADAM10 was increased by elevated levels of ADAM10 mRNA in urinary sediment of the patients with type 2 diabetic mellitus [44]. Therefore, we presume that these metalloproteinases may be involved in the development of glomerular kidney diseases and can serve as new, early biomarkers for glomerular injury in diabetes.

#### 5.2.2. Cathepsin Proteases

Cathepsin proteases have long been implicated as disease drivers [38]. Regarding the role of cathepsins in the kidney filtration function, it was shown that inhibition of the endopeptidase cathepsin L can reduce experimental proteinuria [45]. Recently, it was also demonstrated that cathepsin L is causally involved in pathogenesis of experimental diabetic nephropathy. Diabetic cathepsin L-deficient mice, programmed to develop diabetes, failed to develop albuminuria and preserved normal renal function [36]. Additionally, cathepsin L was reported to be overexpressed in several other proteinuric kidney diseases, along with increased levels of dynamin, which regulates the actin network and nephrin turnover in podocytes [46]. Moreover, it was demonstrated that dynamin is a potential therapeutic target in CKD [47].

Cathepsin D is important for maintaining podocyte integrity. It was shown that cathepsin D-deficient mice develop slit diaphragm disruption and proteinuria [48]. Moreover, it was proved that significantly higher circulating cathepsin D concentrations are present in newly diagnosed type 2 diabetes [49]. Another study showed that cathepsin D levels were also significantly increased in patients with type 2 diabetes, both with and without diabetic retinopathy [50]. These studies indicate that cathepsin D might serve as a potential marker in type 2 diabetes. The presence of cathepsin C in podocytes, as well as its secretion into extracellular space, was recently demonstrated. Moreover, this study confirmed that elevated cathepsin C expression in podocytes correlates with increased glomerular albumin permeability. Cathepsin C expression and activity were also increased in the urine of type 2 diabetic rats. Additionally, cathepsin C deletion was shown to markedly ameliorate nephrin and GLUT4 expression in podocytes cultured in a hyperglycemic milieu, which may improve insulin sensitivity in these cells [34].

#### 5.2.3. Calpain

Another cysteine peptidase, calpain, is an essential interaction partner of the transient receptor potential cation channel 6 (TRPC6) that regulates podocyte motility and adhesion to the GBM [51]. TRPC6 interacts with the podocyte-specific proteins nephrin and podocin, which have both been shown to regulate its activity and localization. Moreover, TRPC6 activity has been linked to increased calpain and calcineurin activity, leading to podocyte injury [52].

It was also demonstrated that augmented TRPC6 expression induced a decrease of the nephrin level in podocytes cultured under hyperglycemic conditions and correlated with increased albuminuria [53].

#### 5.2.4. Urinary Activity of DPP-4

Dipeptidyl peptidase 4 (DPP-4) has been shown to be expressed in podocytes in patients with DKD, but not in healthy individuals. Furthermore, treatment with a DPP-4 inhibitor was found to delay the exacerbation of damage, due to diabetic nephropathy, in a glucose-independent manner [54]. 

Elevated expression and urinary activity of DPP-4 have also been linked to renal fibrosis in DKD [55].

Alterations in the concentration and activity of various proteases in urine have additionally been reported in diabetic patients and experimental animal models. Some of them are proposed to serve as potential biomarkers of renal injury in diabetes. These include cathepsin B, cathepsin D, kallikrein 4, and DPP-4 [56]. Additionally, several members of the cathepsin family (cathepsins A, C, and D), neprilysin, and neutrophil elastase were also found in urine exosomes in a large-scale proteomic analysis [57].

#### 5.2.5. Caspase

In the last decade, the cysteine-aspartic protease (caspase) system has been commonly used to monitor glomerular injury, particularly in in vitro studies. It was confirmed that caspases 3 and 9 are activated in glucose-stressed podocytes, implying that they are agents of podocyte apoptosis in DN [58]. Another study demonstrated that caspase-1-dependent inflammasome activation in podocytes plays a crucial role in the establishment of DKD [59]. In addition to a range of proteolytic enzymes, podocytes also express protease-activated receptors (PARs) 1–4 [60].

#### 5.2.6. Thrombin

Thrombin is a serine protease that regulates the glomerular filtration rate and renal hemodynamics via PARs. It was demonstrated that elevated urinary thrombin is associated with glomerulonephritis and leads to PAR overstimulation, increased intracellular calcium concentration, and proteinuria. Moreover, the inhibition of thrombin was shown to reduce albuminuria in two rat nephrosis models [37]. These studies underline the importance of the distinction of the roles of different proteases. Although essential proteases and their targets have been identified, it is still largely unclear which particular podocyte proteins are cleaved and how proteolytic cleavage affects their functions in physiological and pathological conditions. At this moment, the function of the podocyte protease network is just beginning to be discovered, especially in the context of the novel potential biomarkers.

**Table 1:** Characteristics of potential DKD biomarkers.

Biomarker	Change in Biomarker Level	Research Model/Localization	Preclinical and Morphological Effects	References
α-Klotho	↓	CKD patients with various stages of disease	Serum concentration and urinary excreted Klotho correlates with eGFR in patients with various stages of CKD.	[29]
	↓	Serum of CKD patients with various stages of disease	Circulating α-Klotho levels were lower in people with reduced kidney function and independently associated with eGFR in patients with CKD.	[30]
	↓	Serum of patients with type 2 diabetic with stage 2–3 CKD	Reduced blood Klotho concentration is associated with increased albuminuria. Klotho levels were correlated with FGF23, vitamin D, and insulin resistance, suggesting that Klotho levels might be affected by renal function.	[31]
ADAM17	↑	Mesangial cells	Glucose induces activation of ADAM17, regulates profibrotic TGFβ, and causes the ac-cumulation of matrix proteins.	[39]
	↑	Kidney cortex of OVE26 mice with type 1 diabetes	ADAM17 contributes to matrix protein accumulation, through activation of NOX4 subunits of NADPH oxidase.	[39]
ADAM10	↑	Urinary podocytes from patients with glomerular diseases	Urinary podocytes mainly expressed the mature form of ADAM10.	[43]
	↑	Urine of patients with various glomerular diseases	Patients with high amounts of vesicular ADAM10 demonstrated lower levels of CD9.	[43]
	↑	Urinary podocytes from patients with type 2 DM	A significant correlation of urinary ADAM10 with urinary advanced glycation end-products.	[44]
Cathepsin L	↑	STZ-induced diabetes in WT and cathepsin L-deficient mice	Cathepsin L-deficient mice fail to develop albuminuria and show better renal function after induction of experimental DN.	[36]
Cathepsin D	↑	Serum and plasma from patients with newly diagnosed type 2 DM	Circulating cathepsin D levels were positively correlated with BMI, triglyceride, HbA1c, and fasting glucose.	[49]
Cathepsin C	↑	Podocyte	Cathepsin C deletion ameliorate nephrin and GLUT4 expression in podocytes cultured in hyperglycemic milieu.	[34]
	↑	Urine and glomeruli from Zucker diabetic fatty rats	Cathepsin C expression and activity were corelated with albuminuria.	[34]
Calpain	↑	Urine and snap-frozen kidney of patients with FSGS	Increased activity of calpain in patients with FSGS was accompanied by a decreased cortical and glomerular talin-1 expression.	[52]
	↑	Podocyte	TRPC6 activity has been linked to increased calpain and calcineurin activity, leading to podocyte injury.	[52]
Dipeptidyl peptidase 4	↑	Kidney from Zucker diabetic fatty rats	Less glomerular and tubulointerstitial lesions after administration with DPP4 inhibitor (sitagliptin).	[55]
	↑	STZ-induced diabetic rats	Inhibition of DPP-4 decreased proteinuria, albuminuria, urinary albumin-to-creatinine ratio, and improved creatinine clearance.	[55]
Caspase-3/-9	↑	STZ-induced diabetes in TMEM16A-/-mice.	Upregulation of TMEM16A induced the activation of apoptosis via increase level of caspase-3/-9.	[58]
	↑	Podocyte	TMEM16A exacerbate renal injury caused by podocyte apoptosis via induction caspase-3/-9.	[58]
Caspase-1	↑	db/db (Lepr db/db), nondiabetic control db/m, Casp1-/-mice	Caspase-1-dependent inflammasome activation has a crucial function in the establishment of diabetic nephropathy.	[59]
Thrombin	↑	Podocyte and urine isolated from Wistar rats	Elevated urinary thrombin is associated with glomerulonephritis and leads to PAR overstimulation, increased intracellular calcium concentration, and proteinuria.	[37]

**Table 2:** Classification of biomarkers, according to the localization and function.

Glomerular Biomarker	Tubular Biomarker	Inflammatory Biomarker
α-Klotho	αklotho	α-Klotho
ADAM10/ADAM17	ADAM10/ADAM17	Caspases
Cathepsins	Dipeptidyl peptidase 4	Thrombin
Calpain	miRNA	Cathepsins
Dipeptidyl peptidase 4		miRNA
Thrombin		
miRNA		

### 5.3. Circulating Micrornas

MicroRNAs (miRNAs) are a group of small (18–22 nucleotides), non-coding sequences that post-transcriptionally regulate gene expression. Their major function is the direct, post-transcriptional suppression or cleavage of mRNA targets, resulting in destabilization of the transcripts. As a consequence, miRNA functions as a critical regulator of many cellular processes [61]. Recent analysis showed that circulating miRNAs are useful as biomarkers of DKD. Several studies have measured urinary and serum miRNA in participants with types 1 and 2 diabetes, in relation to different DKD stages [62,63,64,65,66,67] (Table 3). However, inconsistent results have been shown on miRNAs as biomarkers, largely due to the various study designs, small sample size, and different types of miRNAs that were analyzed. Taken all together, further validation studies are necessary to identify specific miRNAs as clinically useful in the prediction of DKD progression.

**Table 3:** Expression of selected microRNAs in patients with diabetic kidney disease.

MicroRNAs	Type of Diabetes (TD)	Sample	Results	Reference
miRNA-29a	T2D	urine	Increased expression level of miRNA-29a was associated with increased albuminuria.	[62]
miRNA-323b-5p	T1D	urine	Decreased in patients with moderately increased albuminuria.	[63]
miRNA-429	T1D	urine	Increased in patients with moderately increased albuminuria.	[63]
miRNA-221-3p	T1D	urine	MicroRNA decreased in patients with severely increased albuminuria.	[63]
miRNA-29b-1-5p miRNA-141-3p miRNA-335-5p miRNA-424-5p miRNA-486-3p miRNA-552 miRNA-619 miRNA-1224-3p miRNA-1912	T1D	urine	MicroRNAs increased in patients with severely increased albuminuria.	[63]
miRNA-320c	T2D	urine	MicroRNA strongly up-regulated in urinary exosomes.	[64]
miR-15b miR-34a miR-636	T2D	urine	MicroRNAs upregulated in both urine pellet and exosome.	[65]
miR-126	T1D	serum	Decreased in patients with increased albuminuria.	[66]

## 6. Implications for Nephroprotection and Therapeutic Strategies

With the very high prevalence of DKD among diabetic patients, as well as the additive impact of DM and CKD on cardiovascular outcomes, attention should be directed towards the possibility of early diagnosis and induction of therapies that have a potential to slow down the progression of kidney failure in the course of diabetes.

The current therapeutic strategies announced by the American Diabetes Association in “Standards of Medical Care in Diabetes—2022” are based on classic DKD markers, such as albuminuria and GFR reduction. These two parameters have a well-documented role in renal failure prognosis estimation; however, they are also laden with downsides, such as the diagnosis delay. The current state-of-the-art in the treatment of DKD concentrates on optimized glucose control, use of sodium-glucose cotransporter 2 (SGLT-2) inhibitors in patients with eGFR ≥ 25 mL/min/1.73 m^2^ and albuminuria (uACR ≥ 300 mg/g), or mineralocorticoid receptor antagonist finrenon in those not capable of receiving SGLT-2 inhibitors, renin–angiotensin–aldosteron (RAA) blockade in moderately albuminuric patients (uACR 30–299 mg/g) [68]. Additionally, RAA blockade is not recommended in normotensives and those who remain undetectable for albuminuria.

This raises the question of the possible role of early markers of DKD, their impact on earlier therapeutic decisions, and the implications for renal function prognosis improvement. At this point, we can only hypothesize how the above-mentioned therapeutic guidelines would change if we had earlier indications to start therapies with proven positive effects on DKD. This would help clinicians to individualize therapy, in order to customize it for patients with a higher risk of renal involvement. Such individualized therapy, at this moment, would consist of several groups of therapeutics that have been proven to slow down CKD progression. Among those, first are SGLT-2 inhibitors that have been shown to exert such effects, regardless of glycemic control [69,70]. Renal benefits and safety have been shown for dapagliflozin, and similar data is anticipated for empagliflozin in 2022 [71,72]. Secondly, GLP-1 receptor agonists have also been shown, with the ability to slow down CKD progression [73]. Finally, there are RAA blocking therapies with long-established roles of ACE inhibitors and AT-1 blockers, as well as an impact on reducing albuminuria and preserving renal function. This group has been recently joined by the anti-aldosterone mineralocorticoid receptor antagonist, finerenone [74]. Finally, a customized therapy consisting of two or three of the above-mentioned therapeutics (e.g., SGLT-2 inh.+GLP-1RA±finerenone) may appear as an effective therapeutic strategy for DKD; this, however, requires further investigations to aggregate evidence based knowledge in the field. 

At the moment, further data from ongoing and planned investigations, designed to compare knowledge from research and clinical trials, is necessary to narrow down the list of potential DKD biomarkers. The future of our patients is just around the corner, and so are the new markers of DKD.

## Data Availability

Not applicable.
